# Mechanical Circulatory Support as a Bridge to Recovery in Fulminant Myocarditis

**DOI:** 10.1186/1749-8090-10-S1-A333

**Published:** 2015-12-16

**Authors:** Yassir Iqbal, Saifullah Mohammed, Aaron Ranasinghe

**Affiliations:** 1 Department of Cardiac Surgery, Queen Elizabeth Hospital, Birmingham, B15 2TH, UK

## Background/Introduction

Ventricular assist devices have been routinely used in patients with end stage heart failure as a bridge to transplantation. We present a case of using a Bi-Ventricular Assist Device (BiVAD) in a patient with fulminant heart failure as a bridge to recovery.

## Aims/Objectives

A 36 year old lady with a history of Grave's Disease and three months post-partum presented with fulminant myocarditis.

She presented to her local hospital on 2nd January with episodes of ventricular tachycardia (VT) and syncope. She was transferred to a tertiary level cardiology centre as she failed to cardiovert. Echocardiography demonstrated significant left ventricular (LV) thickening and LV stasis. She continued to have VT storms and was referred to our advanced heart failure service.

## Method

On arrival she rapidly deteriorated with recurrent VT and ventricular fibrillation (VF) episodes causing severe haemodynamic compromise. A decision was made to institute mechanical circulatory support with veno-arterial Extra-Corporeal Membrane Oxygenation (VA- ECMO). During cardiopulmonary resuscitation she was placed onto VA-ECMO and stabilised. That same evening she underwent conversion to BiVAD support for offloading of both ventricles.

## Results

She was extubated on the first post-operative day. Myocardial biopsy performed during BiVAD implantation confirmed florid myocarditis.

Over the ensuing days, she was mobilised and rehabilitated on the ITU and there was a gradual recovery of myocardial function. On the 25th post-operative day her BIVAD circuit was explanted following a successful wean.

After further rehabilitation she was discharged home, 39 days after admission. MRI prior to discharge demonstrated satisfactory biventricular function with a small right atrial thrombus.

## Discussion/Conclusion

Ventricular assist device therapy can be utilised as an effective primary treatment modality for patients in fulminant heart failure within the spectrum of post-partum cardiomyopathy. We suggest early discussions with regional advanced heart failure centres to facilitate prompt intervention leading to full recovery.

